# The future regulation of artificial intelligence systems in healthcare services and medical research in the European Union

**DOI:** 10.3389/fgene.2022.927721

**Published:** 2022-10-04

**Authors:** Janos Meszaros, Jusaku Minari, Isabelle Huys

**Affiliations:** ^1^ Division of Clinical Pharmacology and Pharmacotherapy, Department of Pharmaceutical and Pharmacological Sciences, KU Leuven, Leuven, Belgium; ^2^ Centre for IT and IP Law (CiTiP), KU Leuven, Leuven, Belgium; ^3^ Uehiro Research Division for iPS Cell Ethics, Center for iPS Cell Research and Application (CiRA), Kyoto University, Kyoto, Japan

**Keywords:** GDPR–General Data Protection Regulation, artificial intelligence, AI Act, healthcare, medical research, data protection, automated decision-making, European Union

## Abstract

Despite its promising future, the application of artificial intelligence (AI) and automated decision-making in healthcare services and medical research faces several legal and ethical hurdles. The European Union (EU) is tackling these issues with the existing legal framework and drafting new regulations, such as the proposed AI Act. The EU General Data Protection Regulation (GDPR) partly regulates AI systems, with rules on processing personal data and protecting data subjects against solely automated decision-making. In healthcare services, (automated) decisions are made more frequently and rapidly. However, medical research focuses on innovation and efficacy, with less direct decisions on individuals. Therefore, the GDPR’s restrictions on solely automated decision-making apply mainly to healthcare services, and the rights of patients and research participants may significantly differ. The proposed AI Act introduced a risk-based approach to AI systems based on the principles of ethical AI. We analysed the complex connection between the GDPR and AI Act, highlighting the main issues and finding ways to harmonise the principles of data protection and ethical AI. The proposed AI Act may complement the GDPR in healthcare services and medical research. Although several years may pass before the AI Act comes into force, many of its goals will be realised before that.

## 1 Introduction

Information technology (IT) companies invest heavily in and cooperate with healthcare organisations to apply their technology in healthcare services and medical research (Corrales Compagnucci et al., 2022). Google (Shetty, 2019) and Apple (Apple, 2021) are present in a growing number of medical fields, from diagnosing cancer to predicting patient outcomes. IBM has made great efforts to apply its artificial intelligence (AI) technology in healthcare by partnering with hundreds of hospitals, healthcare organisations and researchers worldwide to translate data into better care (IBM Watson Health in Oncology, 2020).

Despite the promising results, the proliferation of AI applications in healthcare and medical research faces technological, legal and ethical issues. The main technological issues are the lack of interoperability and standardisation among medical IT systems (Brindha, 2012). From the ethical perspective, healthcare decisions often involve complex judgments and grasping the social context, which AI applications still struggle to replicate or simulate (Louwerse et al., 2005). Reliability and transparency are crucial aspects of building trust in care relationships (Wachter, 2010), and the opaque nature of AI applications might undermine these relationships (Cabitza and Zeitoun, 2019). Moreover, algorithms can underperform in novel cases of drug side effects and underrepresented populations, possibly leading to discrimination (Garcia, 2017).

Building and training AI systems require a vast amount of accurate data, which can contain sensitive medical information in healthcare services and medical research. Therefore, data protection is a critical legal matter, especially in the European Union (EU), under the General Data Protection Regulation (GDPR). The GDPR prohibits solely automated decision-making (ADM) and processing of health data, with a few exemptions, such as if it is done with the patient’s consent or for the public interest. Hence, using health data with AI systems for ADM can face significant legal restrictions. However, the GDPR encourages innovation and technological developments, especially in scientific research, where there are several broad exemptions. Our paper elucidates how these special rules affect the development and application of AI systems in healthcare and medical research.

Nevertheless, the GDPR only partly covers the regulation of AI systems, with rules on processing personal data and protecting data subjects against ADM. It does not provide comprehensive protection against AI systems. Thus, AI regulation has become a central policy question in the EU (European Commission, 2019a), moving from a soft-law approach, with its non-binding guidelines, to a legislative approach that calls for a new regulatory framework on AI by proposing the AI Act. The proposal aims to establish horizontal rules for the development and application of AI-driven products, services and systems in the EU.^1^ With the proposed AI Act, the EU aims to establish a technology-neutral definition of AI systems in EU law and to lay down a classification system for AI systems with different requirements and obligations tailored to a “risk-based approach”.

Given that the interaction between the GDPR and the proposed AI Act may result in a complex legal framework in the future, we elucidate herein the emerging regulatory issues on AI systems in healthcare services and medical research in the EU. We first analyse the legal background of ADM and scientific research in the GDPR. We then introduce and clarify the proposed AI Act regarding healthcare services and medical research. Finally, the article concludes with a novel elaboration on the connection between the principles of data protection and ethical AI.

## 2 Data protection and automated decision-making in healthcare and medical research

Traditionally, health data are collected and processed for specific purposes, such as diagnosis and direct care. Thus, data protection and medical laws worldwide encompass the purpose limitation principle, which means that health data should not be processed for a new purpose, except if certain conditions are met. However, modern healthcare systems and applications, such as AI medical devices, can collect and process a vast amount of health data that can be used for scientific research and policy planning (Vayena and Tasioulas, 2016). In the age of big data and AI, technology provides unprecedented opportunities for the secondary use of health data (Coorevits et al., 2013; see also Corrales Compagnucci, 2019). It would need disproportionate efforts to acquire explicit consent from a large number of data subjects for new processing purposes, which poses complex ethical, legal and technical challenges (Burton et al., 2017). Hence, the purpose limitation principle is increasingly being challenged by researchers and policymakers to provide more efficient care while saving on expenses. Countries must balance citizens’ autonomy, the public interest, and safeguards when healthcare data are reused for secondary purposes to address these challenges (Rumbold and Pierscionek, 2017). The onset of the coronavirus disease 2019 (COVID-19) pandemic became another vital reason to harvest health data to protect public health and address the current pandemic and future ones.

Health data are defined broadly in the GDPR as “personal data related to the physical or mental health of a natural person, including the provision of healthcare services, which reveal information about his or her health status”.^2^ The GDPR generally prohibits processing sensitive data, such as health data.^3^ However, it provides several exemptions from this prohibition, including the case of public health emergencies during the COVID-19 pandemic. These exemptions include when “processing is necessary for reasons of public interest in the area of public health, such as protecting against serious cross-border threats to health”^4^ or when “necessary for reasons of substantial public interest”.^5^ The most practical legal basis for private companies’ processing of data is the data subjects’ consent or a legitimate interest.^6^ For governments, public interest might be a more appropriate legal basis than the data subjects’ consent. The European Data Protection Board has emphasised that consent is not the optimal basis of public authorities’ processing of data due to the power imbalance between the citizens and the authorities (European Data Protection Board 2021), which is also true in the context of the COVID-19 outbreak (European Data Protection Board 2020; see also Fedeli et al., 2022).

### 2.1 Profiling and (solely) automated decision-making

The GDPR’s rules on profiling and (solely) ADM have significantly impacted the application of AI systems in healthcare services and medical research. It is crucial to differentiate profiling, ADM, and solely ADM from each other.

The GDPR defines profiling as follows:

Any form of automated processing of personal data consisting of the use of personal data to evaluate certain personal aspects relating to a natural person, in particular to analyse or predict aspects concerning that natural person’s performance at work, economic situation, health, personal preferences, interests, reliability, behaviour, location or movements.^7^

The most important elements of profiling are 1) automated processing and 2) evaluating the personal aspects of a natural person. As Article 29 Working Party highlighted, “evaluating” indicates that profiling may involve assessing or judging a person. A simple classification of people does not constitute profiling.^8^ For instance, when a healthcare provider sorts patients by age or gender without predictions or further assessment, it is not considered profiling. The Council of Europe’s Recommendation^9^ identified three stages of profiling: 1) data collection, 2) automated analysis to identify correlations and 3) identifying the characteristics of present or future behaviour. Therefore, when COVID-19 patients’ electronic health records with automated analysis systems are combined with their current diagnoses to predict the severity of their diseases, it constitutes profiling.

ADM means an automated decision regarding an individual, with meaningful human involvement, whereas “solely ADM” does not have meaningful human involvement and is a decision made exclusively by an algorithm. By contrast, profiling does not involve a decision and can be only a source of both types of ADM (see the examples in Table 1).^10^ The first element of solely ADM is a “decision” (regarding an individual). In this regard, solely ADM affects healthcare services more than medical research because the primary goal of scientific research is producing new knowledge rather than making decisions regarding individuals (Meszaros and Ho 2021). The second element is the “lack of meaningful human involvement”. To qualify as meaningful human involvement, “the controller must ensure that any oversight of the decision is meaningful, rather than just a token gesture. It should be carried out by someone who has the authority and competence to change the decision”.^11^ In healthcare services, a medical professional’s expected level of oversight to reach “meaningful” involvement is still a debated topic. It needs to be more than routine approval to effectively protect patients against the potential errors of AI systems. The third element is “legal effects or similarly significant consequences”, which might significantly affect a person’s legal status or rights. A legal effect requires that the decision affects someone’s legal rights, such as the freedom to associate with others, vote in an election, or take legal action. A legal effect may also affect a person’s legal status or rights under a contract. Entitlement to or denial of a social service also belongs here.^12^ Decisions in healthcare services thus fulfil this condition. The GDPR permits profiling and ADM for data controllers based on specific legal grounds, with appropriate safeguards. However, solely ADM is generally prohibited, with specific exceptions, such as explicit consent or Member State law (see Table 2).

**TABLE 1:** Examples of profiling and (solely) automated decision-making in healthcare services related to COVID-19.

	Examples
**Profiling**	The patient’s COVID-19 diagnosis is combined with her electronic health records (EHR). The AI system creates her health profile to predict the future severity of her disease (e.g., patients with diabetes have an increased chance of severe COVID-19 symptoms)
**Solely automated decision-making**	An AI system decides alone, **without human involvement**, if the COVID-19 patient can leave the hospital
**Automated decision-making**	There is a **meaningful human involvement:** the AI system in the hospital only supports the medical professionals who are making the final decisions

**TABLE 2:** The impact of profiling, automated decision-making and scientific research on the data subjects’ rights in the General Data Protection Regulation (Meszaros, 2022).

	Profiling	Decision-making with profiling	Solely automated decision-making with profiling	Scientific research (no automated decision-making)
**Prohibitions for data controllers**	**Allowed** (based on specific legal grounds)		**General prohibition** (with exceptions)	**Allowed** (based on specific legal grounds)
**Data subjects’ rights**	Right to be informed - data collected directly (Art. 13) and indirectly (Art. 14 (3)) Right of access (Art. 15) Right to rectification (Art. 16) Right to erasure (Art. 17) Right to restriction (Art. 18) Right to data portability (Art. 20) Right to object (Art. 21)			Right to information in the case of **directly** collected data (Art. 13) Right to data portability (Art. 20)

Overall, the GDPR’s prohibition of solely ADM has a significant effect on the application of AI systems in healthcare services, which might be avoided in several ways, such as with meaningful human involvement.

### 2.2 Scientific research in the General Data Protection Regulation

The GDPR has special rules on scientific research, encouraging innovation and technological development in and through such areas.^13^ There are several exemptions from the strict rules in GDPR for scientific research. For instance, personal data can be used further without the data subjects’ consent for research purposes, and the right to erasure (the right to be forgotten) can be rejected. It is not an uncommon practice in scientific research, especially in medical sciences, to process personal data for a purpose different from the original one (i.e., “secondary use” or “further processing”) to pursue new findings (Auffray et al., 2016). The GDPR acknowledges that “it is often not possible to fully identify the purpose of personal data processing for scientific research purposes at the time of data collection”.^14^ This recognition is crucial because it became more difficult to obtain consent under the GDPR as the consent must be unambiguous and specific to the processing operation.^15^ The GDPR, in principle, forbids data controllers from processing sensitive personal data,^16^ and as a general rule, researchers may use sensitive data only with specific legal grounds, such as explicit consent.^17^ However, the GDPR also intends to ease the restrictions on processing sensitive data by explicitly permitting processing for research purposes. To obtain this permission, data controllers must apply appropriate safeguards,^18^ such as de-identification.

The GDPR defines scientific research as “technological development and demonstration, fundamental research, applied research and privately funded research” conducted by both public and private entities.^19^ Furthermore, the GDPR supports technological and scientific developments by citing the Treaty on the Functioning of the European Union to achieve the European Research Area.^20^ However, the GDPR defines scientific research in the recital part, which is not legally binding.^21^ Therefore, the EU Member States can tailor its scope, resulting in a fragmented legal landscape across the EU, which is against the main goal of GDPR. The European Data Protection Supervisor also highlighted the possible misinterpretation of this exemption. For instance, a company doing research may interpret the pertinent provisions in GDPR as allowing the retention of personal data for indefinite periods and denying data subjects’ rights to information (European Data Protection Supervisor, 2020). Due to this broad exemption for research purposes, it is crucial to clarify and harmonise the definition of scientific research and appropriate safeguards at the EU level (Amram, 2020; Ducato, 2020).

### 2.3 The impact of scientific research on data subjects’ rights in the General Data Protection Regulation

The GDPR has a special legal regime for scientific research, which heavily influences the data subjects’ rights. When personal data are processed for scientific research purposes, Union or Member State law may provide for derogations from the rights of access (Article 15), rectification (Article 16), erasure (Article 17) and restriction of such processing (Article 18) and from the right to object (Article 21). These derogations are provided if these rights are likely to render impossible or seriously impair the achievement of the research purposes and if such derogations are necessary for the fulfilment of the research purposes.^22^ However, two rights remain for the data subjects in every case: the right to information and data portability (see Table 2).^23^

With the aforementioned special rules on scientific research, GDPR attempts to balance privacy and the “ethical and scientific imperative” to share personal data for scientific research (Meszaros, 2022). These rules provide robust protection for data subjects. However, the application of AI systems requires a more specific, novel regulation, which the EU aims for with the proposed AI Act.

## 3 The European Union Artificial Intelligence Act proposal

### 3.1 The regulation of artificial intelligence in the European Union

As the GDPR only partly covers the regulation of AI systems, mainly through processing personal data and protecting of data subjects against ADM, it does not provide comprehensive protection against AI systems. The regulation of these systems requires a more complex legal landscape with strict enforcement, especially in healthcare services and medical research. While the EU does not yet have a specific legal framework for AI, the European Commission (EC) highlighted the necessity of using a regulatory approach to promote this emerging technology and address the associated risks (European Commission, 2020). Due to the economic, legal and social implications of AI, in recent years, AI regulation has become a central policy question in the EU (European Commission, 2019a).

The EU adopted a soft-law approach with its non-binding Ethics Guidelines for Trustworthy AI (European Commission, 2019b) and Policy and Investment Recommendations in 2019 (European Commission, 2019c). However, with the publication of Communication on Fostering a European Approach to Artificial Intelligence (European Commission, 2021) in 2021, the EU shifted towards a legislative approach and called for a new regulatory framework on AI.

The EU unveiled a proposal for the AI Act in April 2021. The legislation would lay down a harmonised legal framework for developing and applying AI products and services. The AI Act aims to ensure that the AI systems made available in the EU market are safe, respect EU law, and provide legal certainty to facilitate investment and innovation in AI. The act seeks to facilitate the development of a single market for lawful, safe and trustworthy AI applications and prevent market fragmentation.^24^ By comparison, it took GDPR more than 4 years from the proposal stage to be adopted, with a 2-year implementation period before it came into force. Although several years may pass before the proposed AI Act comes into force, similar to what happened with GDPR, many of its goals may be realised before that, in healthcare services and medical research.

### 3.2 Definition of artificial intelligence

There is no precise, globally accepted definition of AI. According to the High-Level Expert Group on Artificial Intelligence (AI HLEG),^25^ AI is a scientific discipline that includes several approaches and techniques, such as machine learning (ML), reasoning, and robotics.^26^ To ensure legal certainty, the EC aims to define AI more clearly in the proposed AI Act as a “software that is developed with [specific] techniques and approaches^27^ and can, for a given set of human-defined objectives, generate outputs such as content, predictions, recommendations or decisions influencing the environments they interact with”.^28^ This broad definition covers AI systems that can be used on a standalone basis and those that can be used as product components.

Annex 1 of the AI Act proposal lists the techniques and approaches used to develop AI. Similar to the UNESCO’s Recommendation on the Ethics of Artificial Intelligence, the proposed AI Act defines “AI system” as a range of software-based technologies that encompasses “machine learning”, “logic and knowledge-based” systems and “statistical” approaches (UNESCO, 2021). ML is a branch of AI and computer science which focuses on using data and algorithms to imitate how humans learn, gradually improving its accuracy.^29^ ML methods are applied in various fields of science, leading to more evidence-based decision-making. Deep learning is a family of ML models based on deep convolutional neural networks (Schmidhuber, 2015). These techniques are gaining popularity because they may achieve human-level performance in various medical fields (LeCun et al., 2015), such as detecting skin cancer (Esteva et al., 2017) and diabetic retinopathy (Ting et al., 2017). The EU plans to update Annex 1 with new approaches and techniques as these emerge, providing flexibility to the proposed AI Act.

### 3.3 Risk-based approach

The proposed AI Act will introduce a risk-based approach to regulating AI systems. With this solution, the legal intervention is tailored to different risk levels, distinguishing between 1) unacceptable risk, 2) high risk, 3) low or minimal risk.

#### 3.3.1 Prohibited risk

The proposed AI Act explicitly bans harmful AI practices considered threats to people’s safety, livelihoods and rights. Accordingly, it prohibits making the following available in the EU market or putting them into service or using them in the EU: 1) AI systems that deploy harmful manipulative “subliminal techniques”; 2) AI systems that exploit specific vulnerable groups (e.g., those with physical or mental disabilities); 3) AI systems used by public authorities or on their behalf for social-scoring purposes and 4) “real-time” remote biometric identification systems in publicly accessible spaces for law enforcement purposes, except in a limited number of cases.

In the context of using health data, “social scoring” may have relevance.^30^ In essence, social scoring means using an AI system to evaluate the trustworthiness of individuals based on their behaviours or personal characteristics, leading to the detrimental or unfavourable treatment of an individual or a group of people.

From a medical perspective, an existing medical condition (e.g., mental disorder) may form a base for predictive social scoring. The relationship with healthcare authorities and adherence to public health measures may also be factors for social scoring, such as following quarantine measures or receiving vaccinations. As social scoring is an unacceptable risk, the EU aims to prohibit using AI for such purposes.

Detrimental or unfavourable treatment might be in a different social context and unrelated to the contexts in which the data were originally generated or collected. For instance, a person guilty of tax evasion cannot use public transport or some public health services due to social scoring. This unfavourable treatment would be unjustified or disproportionate.

#### 3.3.2 High-risk artificial intelligence systems

The proposed AI Act lists high-risk AI systems in the eight specific areas below.

(1) Biometric identification and categorisation of natural persons: This may be crucial in healthcare services, such as for identifying and sorting patients in a hospital based on their medical history and appointments.
(2) Management and operation of critical infrastructure: This may include the software for managing public healthcare services and electronic health records.
(3) Education and vocational training: AI systems will also affect the education of medical professionals. Students need to learn about AI products and services and prepare to use them due to their current proliferation in healthcare services and medical research.
(4) Employment, worker management and access to self-employment: The workforce in both public and private health services and research institutes may be affected by this future regulation.
(5) Access to and enjoyment of essential private and public services and benefits: As both public and private health services are mentioned here, the proposed AI Act may have a crucial impact on these fields.
(6) Law enforcement
(7) Migration, asylum and border control management
(8) Administration of justice and democratic processes

The list of high-risk AI systems in the annexe of the proposed AI Act provides flexibility for the EU as it can be modified and expanded in the future.^31^ There are several requirements for these high-risk AI systems, such as risk management and data governance.^32^ The providers of these systems are required to register their systems in an EU-wide database before making them available in the market or deploying them into service. However, several types of AI products already fall under conformity assessment, such as medical devices. These products remain under their current assessment framework.

#### 3.3.3 Low- and minimal-risk at systems

Low- or minimal-risk AI systems can be developed and used in the EU without conforming to any additional legal obligations. However, the proposed AI Act envisages the voluntary creation of codes of conduct to provide safe and reliable services. Examples of these AI systems are those interacting with humans (e.g., chatbots) and provide emotional recognition. These tools may help interact with patients in healthcare services and participants in medical research.^33^

## 4 Discussion and actionable recommendations

To realise AI’s potential in healthcare and medical research, new laws regulating AI systems are necessary (Humerick, 2018), based on the existing guidelines and harmonised with GDPR. The proposed AI Act is a crucial step herein. However, harmonisation with GDPR is an essential legal issue that needs to be discussed. AI HLEG^34^ has laid down the most important principles of ethical AI. We expand these principles into the healthcare context and elaborate on their connection with the GDPR’s data protection principles, providing a novel perspective. Our goals are to highlight the critical issues on AI in healthcare and to provide recommendations for applying GDPR and the proposed AI Act in the future.

(1) Technical robustness and safety: To prevent or minimize the probability of unintentional harm, AI applications in healthcare and research need to be secure and resilient. Technical robustness also means ensuring a fallback plan in case something goes wrong and being accurate, reliable and reproducible. The GDPR and the proposed AI Act require technical robustness and safeguards for processing personal data and deploying AI systems.^35^ However, both do not detail these safeguards due to the rapidly changing technological environment, providing “future-proof” regulation. The necessary safeguards, such as “pseudonymisation”, differ among the EU Member States (Meszaros and Ho, 2018). Therefore, the required safeguards and the review process by authorities need harmonisation, especially in the case of AI systems for healthcare services and medical research (Malgieri, 2019).

The proposed AI Act provides two types of conformity assessments depending on the AI system: self-assessment and assessment by notified bodies. Regarding self-assessment, the developer of an AI system is responsible for compliance with the requirements on quality and safety. When the assessment is conducted by a notified body, an independent third party certifies the AI system’s compliance. However, the review process by notified bodies needs to be harmonised in the EU, otherwise, the developers of AI systems will opt for the less strict notified bodies, resulting into forum shopping.

(2) Privacy and data governance: There is a complex connection between the GDPR and the proposed AI Act. They may complement each other and share definitions related to data protection, such as their rules on biometrics and special categories of data.^36^ The AI Act clarifies that it should not be understood as providing legal grounds for processing personal data, including special categories of personal data.^37^ Therefore, in general, the AI Act does not provide a legal basis for the primary or secondary use of personal data, especially those under special categories, such as health data.

However, there are exemptions from the above rule, such as the concept of a “regulatory sandbox”. A “regulatory sandbox” is a “safe space in which businesses can test innovative products, services, business models and delivery mechanisms without immediately incurring all the normal regulatory consequences of engaging in the activity in question” (Financial Conduct Authority, 2015). Regulatory sandboxes were first used within the financial technologies (FinTech) sector but have expanded into other sectors, including healthcare (Leckenby et al., 2021; see also Fenwick et al., 2018). The AI Act will provide a legal basis for processing personal data for developing certain AI systems in the public interest within the AI regulatory sandbox, in line with the GDPR.^38^

(3) Human agency and oversight: These are essential, especially in high-risk AI systems. Human oversight has a central role in the proposed AI Act,^39^ which states that it “will also facilitate the respect of other fundamental rights by minimising the risk of erroneous or biased AI-assisted decisions in critical areas”. As we previously highlighted, the GDPR’s restrictions on solely ADM can be avoided with meaningful human involvement. However, to qualify as having meaningful human involvement, “the controller must ensure that any oversight of the decision is meaningful, rather than just a token gesture” and “it should be carried out by someone who has the authority and competence to change the decision”.^40^ Overall, proper oversight is necessary, especially in the case of AI medical devices and applications, for patient and research participant safety.
(4) Transparency: Transparency is one of the data-processing principles in GDPR,^41^ which prevails through several rights, such as the right to access and be informed.^42^ In the proposed AI Act, transparency is required for specific AI systems, such as high-risk ones. In healthcare services and medical research, decisions need to be transparent and explainable for safety and trust. Furthermore, scientific research aided by AI applications should be transparent for reproducibility and inquiries about bias and safety.
(5) Diversity, non-discrimination and fairness: The data used to train AI systems need to be diverse to avoid bias. This requirement is of utmost importance in the case of AI systems because they might cause harm to populations underrepresented in healthcare. Therefore, one of the aims of the AI Act proposal is to “minimise the risk of algorithmic discrimination, in particular concerning the design and the quality of data sets used for the development of AI systems complemented with obligations for testing, risk management, documentation and human oversight throughout the AI systems’ lifecycle”.^43^
(6) Accountability, societal and environmental well-being: As highlighted by AI HLEG, mechanisms should be put in place to ensure responsibility and accountability for AI systems and their outcomes.^44^ Certain actors, such as the government, IT, or special insurance companies, should be held responsible for the unintended consequences of these services. Finally, when AI is used for healthcare and research, it is crucial to use it transparently to benefit the whole society by respecting democratic values and decisions.

Overall, the black-box nature of AI applications and devices cannot be an excuse for complying with privacy and safety regulations. The proposed AI Act also highlights that it complements the GDPR without prejudice.^45^ These two regulations can be the main pillars of safety and innovation in AI systems for healthcare and medical research.

## 5 Conclusion

The GDPR’s prohibition of solely automated decision-making significantly effects the application of AI systems in medical research and healthcare services. While in medical research, the main focus is on innovation and efficacy, in healthcare services (automated) decisions are made frequently, even rapidly. Therefore, the GDPR’s restrictions on solely automated decision-making apply mainly to healthcare services. Hence, the rights of patients and research participants may differ significantly.

The proposed AI Act introduced a risk-based approach to AI systems based on the principles of ethical AI. We highlighted the complex connection between the GDPR and the proposed AI Act. For instance, they may complement each other and share the same definitions related to data protection. In some cases, the AI Act may provide a legal ground for processing personal data. Human agency and oversight must also be harmonised, especially the expectations of meaningful human involvement, in connection with the GDPR’s rules on solely automated decision-making.

The current and future regulation of AI and data protection in the EU need to align well to provide a safe and innovative future. Although several years may pass before the proposed AI Act comes into force, many of its goals may start being realised before that. Harmonising the data protection principles and ethical AI is a complex but desirable goal, especially in healthcare services and medical research.

## Footnotes

1 Proposal for a regulation laying down harmonised rules on artificial intelligence (Artificial Intelligence Act), Brussels, 21.4.2021, COM (2021) 206 final.

2 GDPR Article 4 (15).

3 GDPR Article 9.

4 GDPR Article 9(i).

5 GDPR Article 9(h).

6 GDPR Article 6 and 9.

7 GDPR Article 4 (4).

8 Article 29 (Working Party). Guidelines on automated individual decision-making and profiling for the purposes of Regulation 2016/679 (2018) 7.

9 Council of Europe. The protection of individuals with regard to automatic processing of personal data in the context of profiling. Recommendation CM/Rec (2010)13 and explanatory memorandum.

10 Ibid.

11 Article 29 (Working Party). Guidelines on automated individual decision-making and profiling for the purposes of Regulation 2016/679 (2018) 21.

12 Ibid 21.

13 The GDPR Recital 157 also highlights that “By coupling information from registries, researchers can obtain new knowledge of great value with regard to widespread medical conditions such as cardiovascular disease, cancer and depression”.

14 GDPR Recital 33 and 65.

15 GDPR Article 4 (11).

16 GDPR Article 9 (1).

17 GDPR Article 9 (1) (a).

18 GDPR Article 9 (2) (j).

19 GDPR Recital 159.

20 Treaty on the Functioning of the European Union, Article 179 (1). The Union shall have the objective of strengthening its scientific and technological bases by achieving a European research area in which researchers, scientific knowledge and technology circulate freely, and encouraging it to become more competitive, including in its industry, while promoting all the research activities deemed necessary by virtue of the other chapters of the treaties.

21 In the EU law, a recital is part of the text, usually the beginning of the law, which explains the reasons for the provisions, and it is not normative, thus legally not binding. Recitals are usually general statements. The GDPR Recital gives guidelines for understanding the normative text and its purposes.

22 GDPR Article 89.

23 GDPR Articles 13 and 20.

24 Proposal for a regulation laying down harmonised rules on artificial intelligence (Artificial Intelligence Act) and amending certain Union legislative acts (COM (2021) 206), Explanatory Memorandum and Recitals 1 and 5.

25 The High-Level Expert Group on Artificial Intelligence was tasked by the European Commission to provide advice on its artificial intelligence strategy.

26 54 High-Level Expert Group on Artificial Intelligence. A definition of AI: Main capabilities and scientific disciplines (2019), p. 8.

27 Listed in Annex 1 of the AI Act.

28 AI Act, Article 3 (1) and Recital 6.

29
https://www.ibm.com/cloud/learn/machine-learning [Accessed June 11, 2022].

30 AI Act proposal, Article 5(c): Social scoring means the “… use of AI systems by public authorities or on their behalf for the evaluation or classification of the trustworthiness of natural persons over a certain period of time based on their social behaviour or known or predicted personal or personality characteristics, with the social score leading to either or both of the following: 1) detrimental or unfavourable treatment of certain natural persons or whole groups thereof in social contexts which are unrelated to the contexts in which the data was originally generated or collected; 2) detrimental or unfavourable treatment of certain natural persons or whole groups thereof that is unjustified or disproportionate to their social behaviour or its gravity”.

31 AI Act Articles 7 and 8.

32 AI Act Articles 8–15.

33 AI Act Title IV.

34 Following the launch of its Artificial Intelligence Strategy in 2018, the European Commission appointed a group of 52 experts to provide advice regarding its implementation. The group members were selected through an open selection process and comprised representatives from academia, civil society and industry. https://ec.europa.eu/digital-single-market/en/high-level-expert-group-artificial-intelligence (Accessed October 12, 2020).

35 AI Act, Article 10 (5), GDPR Article 89.

36 GDPR Article 9.

37 AI Act proposal Recital 41.

38 AI Act Recital 72.

39 AI Act Article 14.

40 Article 29. Working Party on Profiling (2018), p. 21.

41 GDPR Article 5 (1)a.

42 GDPR Article 15.

43 AI Act 1.2. Consistency with existing policy provisions in the policy area.

44 High-Level Expert Group on Artificial Intelligence. Ethics guidelines for trustworthy AI (2018).

45 AI Act 1.2. Consistency with existing policy provisions in the policy area.
